# The association of tonsillar microbiota with biochemical indices based on obesity and tonsillar hypertrophy in children

**DOI:** 10.1038/s41598-023-49871-y

**Published:** 2023-12-20

**Authors:** Jiwon Park, Kyeong Eun Lee, Da Hyeon Choi, Yoon-Keun Kim, Won Hee Lee, Min Su Kim, Han Wool John Sung, Jae Won Chang, Yoon Shin Park

**Affiliations:** 1 Department of Biological Sciences and Biotechnology, School of Biological Sciences, College of Natural Sciences, Chungbuk National University, Cheongju, 28644 Republic of Korea; 2 Institute of MD Healthcare Inc., Seoul, 03923 Republic of Korea; 3 Department of Otolaryngology-Head and Neck Surgery, Chungnam National University College of Medicine, Daejeon, 35015 Republic of Korea

**Keywords:** Microbiome, Paediatrics, Body mass index

## Abstract

The correlation between tonsil microbiome and tonsillar hypertrophy has not been well established. Given that oral dysbiosis is related to several metabolic diseases and that tonsillar hypertrophy leads to disordered breathing during sleep and obesity in children, it is necessary to investigate the relationship between the oral microbiome and tonsillar hypertrophy. After 16S rRNA amplicon sequencing of tonsillectomy samples, we evaluated the correlation between the tonsil microbiome and biochemical blood indices in pediatric patients who underwent tonsillectomy. Groups are classified into two categories: based on BMI, and grades 2, 3, and 4 based on tonsil size. Children with obesity and tonsillar hypertrophy have similar microbiome compositions and induce comparable changes in microbiome abundance and composition, confirming the association from a metagenomic perspective. In addition, obesity and tonsillar hypertrophy demonstrated a strong correlation with the Proteobacteria to Firmicutes (P/F) ratio, and among various biochemical indicators, alanine aminotransferase (ALT) levels increase with obesity and tonsillar hypertrophy, indicating a possible association of tonsil microbiome and liver metabolism. These novel findings demonstrate the significance of the tonsil microbiome and suggest the need for tonsil regulation, particularly during childhood.

## Introduction

The oral microbiome is the second largest microbial community in humans after the gut microbiome. Bacterial growth occurs in the human oral cavity in a variety of habitats including the tongue, teeth, hard and soft palates, and the tonsils^1^. Oral bacteria contribute to oral diseases and are a significant risk factor for several diseases affecting other parts of the body, including diabetes^2^, atherosclerosis^3^, and inflammatory bowel disease^4^. The composition of the oral microbiome is influenced by host health and lifestyle-related factors, and the host immune system plays an important role in the homeostasis of the oral microbiome. Thus, oral microbiome dysfunction is associated with several oral and systemic diseases^5^, and occasionally, the oral microbiome can also enhance the host immune system^6^.

Tonsils are connected to both the digestive and the respiratory systems and can be affected by bacteria from the saliva and the digestive tract. We previously investigated the association between the saliva and the tonsil microbiomes and reported similar diversity and composition between the two microbiomes^7^. Obesity, adenoid vegetation, and tonsillar hypertrophy are significant risk factors for developing obstructive sleep apnea (OSA), which adversely affects physical growth^8^, neurocognitive functions^9^, and behavioral functioning^10^ in children. A previous study reported a larger tonsil size in children with obesity and sleep-disordered breathing, with a positive correlation between the palatine tonsil and adenoid size and the apnea–hypopnea index in both children with obesity and those with normal weights^11^. OSA caused by obesity and tonsillar hypertrophy can lead to oral microbiome alterations due to respiratory failure. A recent study demonstrated a correlation between the tonsillar microbiome and chronic tonsillitis and OSA in children, as well as a correlation between the gut microbiome and host weight^12^. Moreover, people with OSA had significantly decreased bacterial diversity compared to people without OSA, with the most significant decrease occurring in people with moderate OSA.

Oral bacteria are substantial risk factors for systemic disorders such as diabetes, preterm birth, and cardiovascular disease, as the oral cavity is a clinically relevant microbial habitat and a gateway to the gut^2,13^. Several studies have shown that the oral microbiome is associated with the development of obesity owing to its modulatory effect on the gut microbiome and that there are differences between the oral microbiomes of obese and non-obese individuals^14,15^. Wu et al*.* reported that the oral microbiome in obese people was significantly lower in diversity and abundance than that in non-obese people, and *Prevotella* and *Granulicatella* genera were considerably higher in the obese group. In contrast, *Haemophilus* and *Corynebacterium* genera were less abundant in the saliva^14^. Another study found that *Actinobacteria* was less prevalent in diabetic patients and obese individuals than in normal-weight individuals^2^. As in Wu’s study, *Haemophilus* was less abundant in the obese and grade 3 and 4 groups, whereas the abundance of *Actinobacteria* did not show any significant difference. However, without a doubt, *Haemophilus* was still the most predominant genus in all groups.

The oral microbial diversity and the ratio of Firmicutes to Bacteroidetes (F/B ratio) correlated with growth curves in 2-year-old children, indicating that the oral microbiota of children with rapid infant weight gain may have an established pattern similar to adults with obesity, suggesting that the oral microbiome could be used to potentially identify the risk of obesity in children^16^. Moreover, another study reported a significant association between the prevalence of oral microbiome-related periodontal disease and obesity in adults^17^.

Although the association between obesity and tonsil hypertrophy that causes obstructive sleep disorder have been established clinically, there was no metagenomic evidence. The microbiome distribution in tonsillar tissues of Korean pediatric patients who underwent tonsillectomy was investigated by 16S rRNA sequencing with and without obesity based on the tonsil grade. To investigate the relationship between tonsillar hypertrophy and the oral microbiome, we compared the distribution and correlation of the microbiome in tonsillar tissues. We evaluated that tonsillar hypertrophy and obesity are associated with the tonsil microbiota, and the possible effect of the tonsillar microbiome on metabolic disorder of the host was identified through the correlation between tonsillar microbiome and biochemical indices. Our findings shed light on the importance of dysbiosis of the tonsil and suggest the need for its regulation, especially during childhood.

## Results

### General characteristics and biochemical indices of the patients

The general and biochemical characteristics of the participants within each group of the two categorical classifications are presented along with the corresponding reference values for Korean children^18,19^ in Table S1. The mean age of the participants was 7.89 ± 2.69 years. Mean anthropometric measurements were: height, 129.65 ± 17.31 cm; body weight, 33.48 ± 15.31 kg; and BMI, 18.95 ± 4.18 kg/m^2^. In category 1, the body weight percentile and BMI of participants who were lean (n = 28) were 41.1 ± 23.5 and 16.4 ± 1.8, respectively, while those of participants with obesity (n = 18) were 95.6 ± 4.6 and 22.9 ± 3.7, respectively. In category 2, body weight percentiles of grade 2 (n = 12), grade 3 (n = 18), and grade 4 (n = 16) patients were 46.3 ± 28.5, 64.1 ± 32.5, and 72.6 ± 32.7, respectively. The BMI of participants in grades 2, 3, and 4 was 16.1 ± 1.7, 19.5 ± 4.2, and 20.5 ± 4.6, respectively. The average values of the biochemical indices of all the participants were within the normal Korean pediatric reference range^20^.

Among the biochemical indices, we observed significantly increased serum ALT levels in the obese group compared to that in the lean group in category 1. Interestingly, the ALT level also positively correlated with increased tonsil size in category 2. The AST/ALT ratios for the entire sample, lean, obese, grade 2, grade 3, and grade 4 groups were 1.85, 2.32, 1.44, 1.98, 1.64, and 1.35, respectively (Table S2).

### Total number of identified and classified microbes by category

We performed 16S rRNA amplicon sequencing based on the V3–V4 hypervariable regions to categorically compare the microbiome of the tonsil samples. A total of 46 samples were processed, and 22 phyla, 48 classes, 106 orders, 222 families, 579 genera, 1 059 species, and 4 778 ASVs were annotated. The taxonomic composition of the microbiota in tonsillar tissue is listed in Table 1. The bacterial taxa with a frequency greater than 25% for each group were selected, in order to remove the low counts of bacterial taxa. In the comparison between the lean and obese groups in category 1, the same number of 11 phylum but more bacterial taxa were annotated in the obese group. In category 2 compared by tonsil grade, bacterial taxa in grades 2 and 3 were at similar levels, but in grade 4, more taxa were annotated. At the genus level, the obese group in category 1 exhibited more species than the lean group, whereas the number of species increased with higher tonsil grade in category 2.

**Table 1:** Analysis of the microbiome by category based on percentile and tonsil grade.

Total number of microbes identified		Phylum	Class	Order	Family	Genus	Species
Category 1	Lean	11	20	29	45	62	111
	Obesity	11	23	33	59	132	197
Category 2	Grade 2	11	23	31	44	61	105
	Grade 3	9	21	30	45	67	115
	Grade 4	14	25	38	66	149	237

Microbiome analysis was performed based on the V3–V4 hypervariable regions of 16S rRNA genes. The total number of microbes was counted as taxa identified at a frequency of more than 25% of each group.

### Comparison between alpha and beta diversities

**Figure 1:**
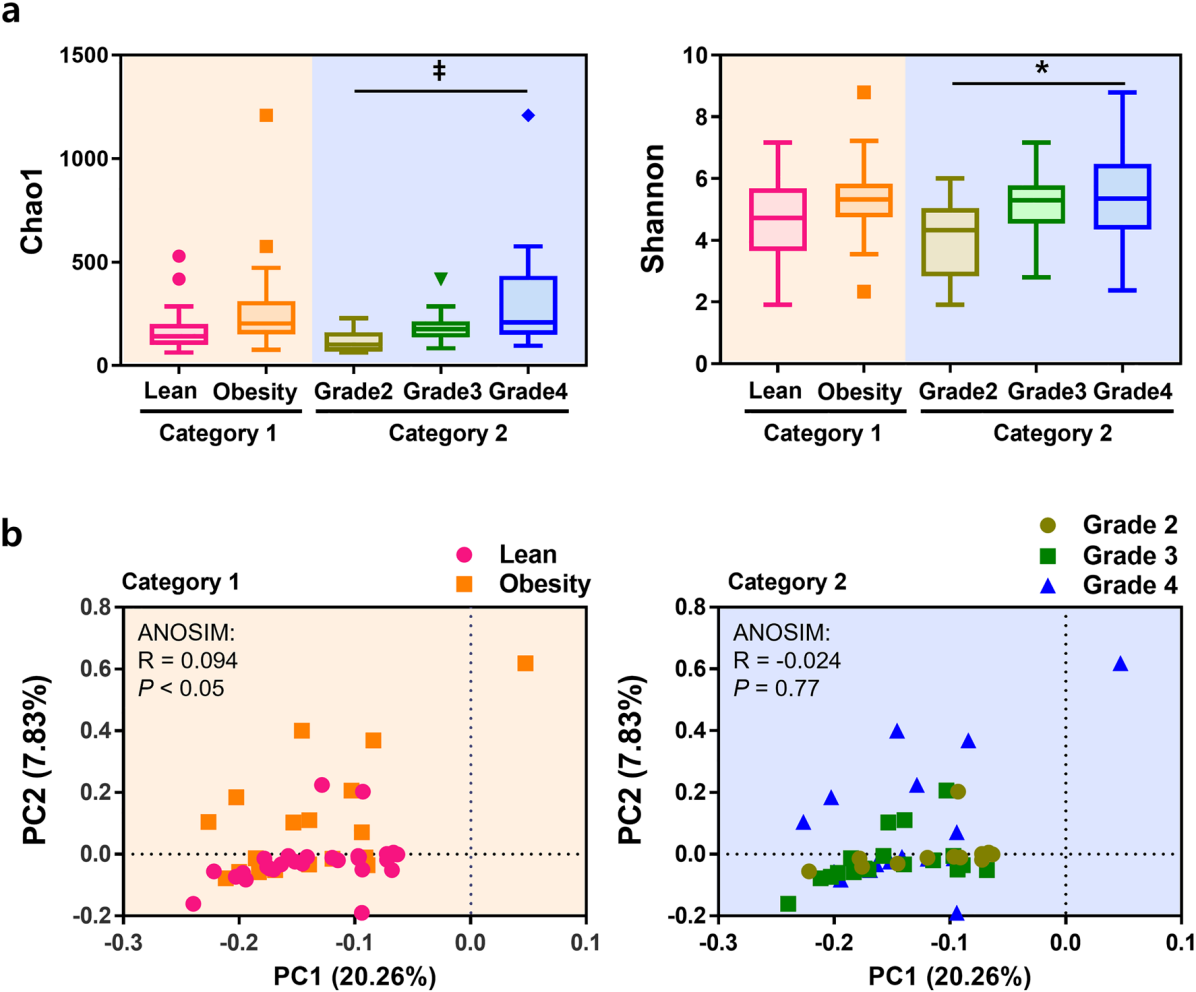
Comparison between alpha- and beta- diversity by category. (**a**) Chao1 and Shannon indices for each category. Statistical analysis was performed using Kruskal–Wallis and Dunn’s post hoc tests (*P < 0.05, ^†^*P* < 0.01, ^‡^*P* < 0.001). (**b**) Beta-diversity-based PCA plots of each category using the Aitchison distance of CLR-transformed counts. Each point represents the sample. One-way ANOSIM was performed to determine the diversity of the microbial communities (Category 1: R^2^ = 0.094, *P* = 0.048, Category 2: R^2^ = − 0.024, *P* = 0.773).

We first assessed the alpha diversity, indicative of microbial abundance, of each group in the two categories according to percentile and tonsil grade. The rarefaction curves for each group are shown in Fig. S1. Chao1 and Shannon indices were used to calculate species diversity by category. In category 1, the Chao1 (*P* = 0.09) and Shannon (*P* = 0.38) indices, representing the richness and evenness of the microbial community, were higher in the obese group than in the lean group (Fig. 1a). In category 2, the alpha diversity indices were higher in grade 3 (Chao1, *P* = 0.08; Shannon, *P* < 0.001) and grade 4 (Chao1, *P* = 0.15; Shannon, *P* = 0.05), compared to grade 2 (Fig. 1a).

The altered microbial composition of each group by category was visualized using PCA of the Aitchison distance of CLR-transformed counts, which was used to compare the categorical phylogenetic composition and to reveal microbial diversity between the groups. Based on PC2 scores, the obese group in category 1 and grade 4 group in category 2 were compared to the other groups (Fig. 1b). Statistical differences between the groups were assessed for significance using ANOSIM of the diversity of microbial communities. ANOSIM revealed a significant difference between the lean and obese groups in category 1 (R = 0.094, *P* = 0.048) and between grade 3 and 4 groups in category 2 (R = − 0.024, *P* = 0.773) (Fig. 1b).

### Microbial composition at the phylum and genus level in tonsillar tissues

**Figure 2:**
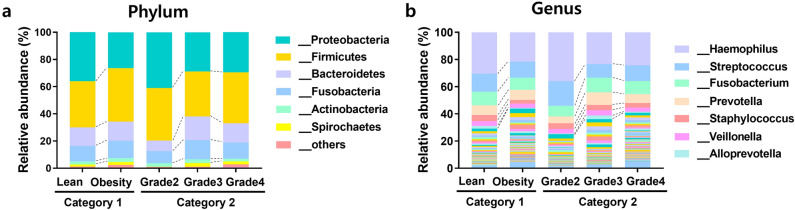
Relative abundance of the microbial community in tonsils. Relative abundance of microbial communities based on dominant phyla and genera. (**a**) The most abundant phyla were Proteobacteria, Firmicutes, Bacteroidetes, Fusobacteria, and Actinobacteria. (**b**) The predominant genera were *Haemophilus*, *Streptococcus*, *Fusobacterium*, *Prevotella*, and *Staphylococcus*.

We determined the microbiome compositions in the tonsillar tissue based on body weight percentiles and tonsil grade groups based on the average relative abundance assigned to the phylum and genus levels (Fig. 2). In general, main phyla of the tonsil were Proteobacteria, Firmicutes, Bacteroidetes, and Fusobacteria in both categories, and these four dominant phyla accounted for more than 90% of total bacteria. The lean group in category 1 showed a high abundance of Proteobacteria (36.0%), followed by Firmicutes (34.1%), Bacteroidetes (13.6%), and Fusobacteria (11.4%) at the phylum level. The obese group contained significantly more Firmicutes (39.3%), while the levels of Proteobacteria (26.4%), Bacteroidetes (14.1%), and Fusobacteria (12.7%) were comparable to those in the lean group. In category 2, Proteobacteria had the highest abundance in grade 2 (40.9%), whereas Firmicutes had the highest abundance in grades 3 (33.2%) and 4 (37.4%). The top ten taxa in both categories are listed in Table 2. At the genus level, *Haemophilus* was the predominant taxon in both categories, but was less abundant in the obese group (21.52%) than the lean group (30.6%), and in grades 3 (23.4%) and 4 (24.4%) than that in grade 2 (36.0%). Six of the top ten ranked taxa were obligate anaerobes. However, the most abundant bacteria in tonsillar tissue were facultative anaerobes such as *Haemophilus*, *Streptococcus*, *Staphylococcus*, and *Gemella*.

**Table 2:** Top 10 microbes in the relative abundance of tonsillar bacterial taxa.

Order	Phylum	Genus	Category 1		Category 2			Gram staining	Aerobic/anaerobic
			Lean (%)	Obesity (%)	Grade 2 (%)	Grade 3 (%)	Grade 4 (%)		
1	Proteobacteria	*Haemophilus*	30.59	21.54	36.06	23.41	24.38	−	Facultative anaerobe
2	Firmicutes	*Streptococcus*	13.14	11.82	18.09	10.01	11.46	+	Facultative anaerobe
3	Fusobacteria	*Fusobacterium*	9.99	8.94	7.80	10.78	9.56	−	Obligate anaerobe
4	Bacteroidetes	*Prevotella*	7.05	7.45	4.93	9.14	6.74	−	Obligate anaerobe
5	Firmicutes	*Staphylococcus*	4.60	2.75	4.30	4.03	3.39	+	Facultative anaerobe
6	Firmicutes	*Veillonella*	3.49	3.19	3.23	3.85	2.94	−	Obligate anaerobe
7	Fusobacteria	*Leptotrichia*	1.15	3.15	0.99	2.89	1.57	−	Obligate anaerobe
8	Bacteroidetes	*Alloprevotella*	2.03	0.51	0.51	2.74	0.66	−	Obligate anaerobe
9	Firmicutes	*Gemella*	1.54	2.84	1.55	2.87	1.51	+	Facultative anaerobe
10	Spirochaetes	*Treponema*	1.50	2.16	0.09	2.63	2.03	−	Obligate anaerobe

The abundance of bacterial taxa in tonsillar tissues detected in each category.

### Comparison of microbial composition by category

**Figure 3:**
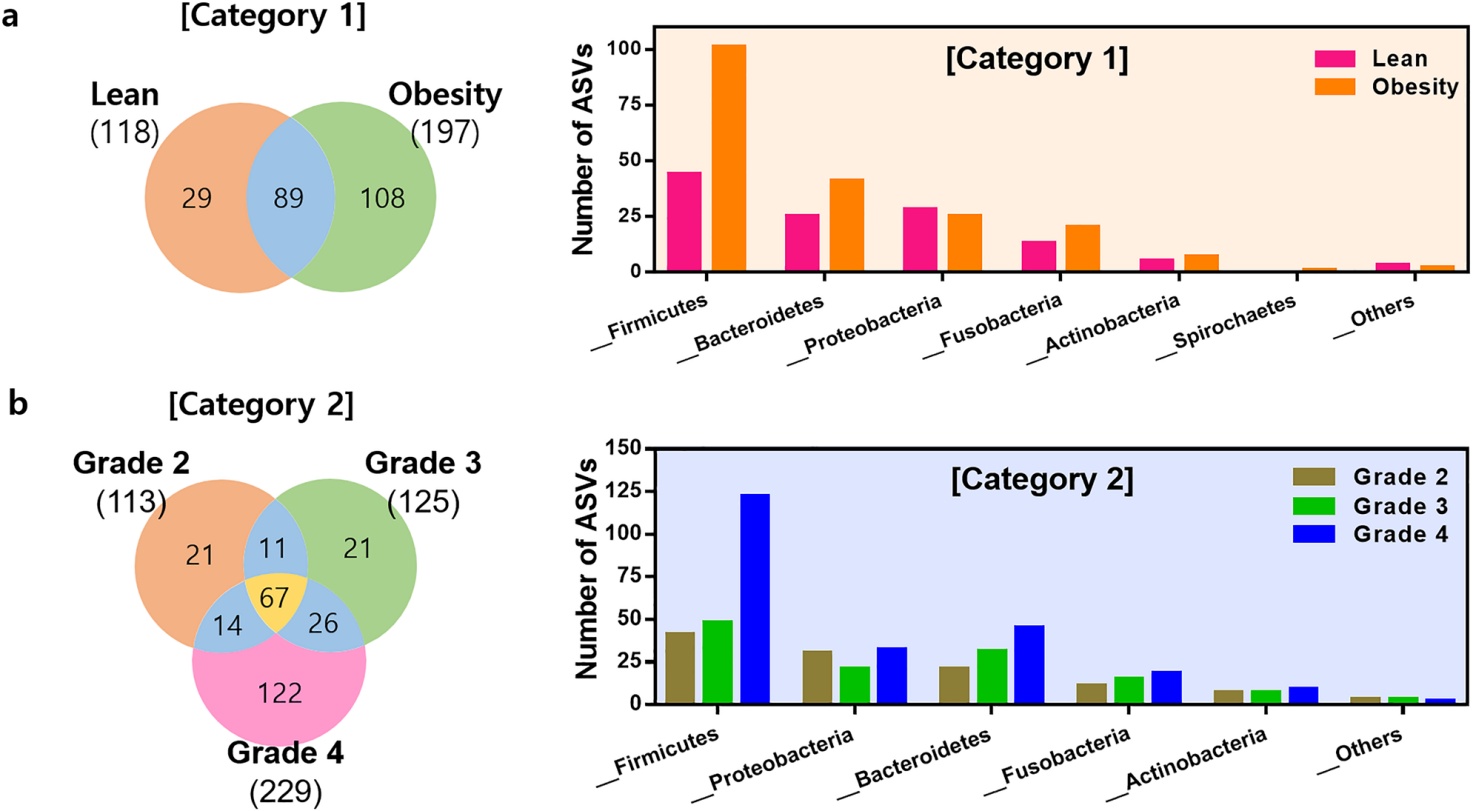
Comparison of bacterial community at the ASV level by category. (**a**) Venn diagram showing the overall number of ASVs overlapping between each group in category 1. More than 90% of all samples and more than 75% of each group with a proportion of zeroes in ASVs were removed for the analysis. The bar plot shows the microbial composition of the ASVs in each group at the phylum level. (**b**) Venn diagram showing the overall number of ASVs overlapping between the grade 2, 3, and 4 groups in category 2. The bar plot shows the microbial composition of the ASVs in each group at the phylum level.

To compare the microbial composition of ASVs between groups within each category, ASVs with a proportion of zeroes in more than 90% of all samples and more than 75% of each group were excluded from the analysis. In category 1, the 118 ASVs in the lean group and 197 ASVs in the obese group are presented as a Venn diagram (Fig. 3a). A total of 89 common ASVs were identified in both groups, corresponding to 75.4% and 45.2% of the lean and obese groups, respectively. *Firmicutes* accounted for a significantly greater proportion in the obese group than in the lean group.

In category 2, the 113 ASVs in grade 2, 125 ASVs in grade 3, and 229 ASVs in grade 4 are presented as a Venn diagram (Fig. 3b). The 67 common ASVs shared by grade 2, 3, and 4 groups corresponded to 59.3%, 53.6%, 29.3% of the groups, respectively. Similar to the obese group in category 1, the composition of *Firmicutes* was the highest in the grade 4 group in category 2. The number of ASVs of *Firmicutes* in the lean, grade 2, and grade 3 groups was comparable, while the number of ASVs in the obese and grade 4 groups was approximately 2.6 ± 0.3-fold.

### Association of microbial communities based on obesity and tonsil size

**Figure 4:**
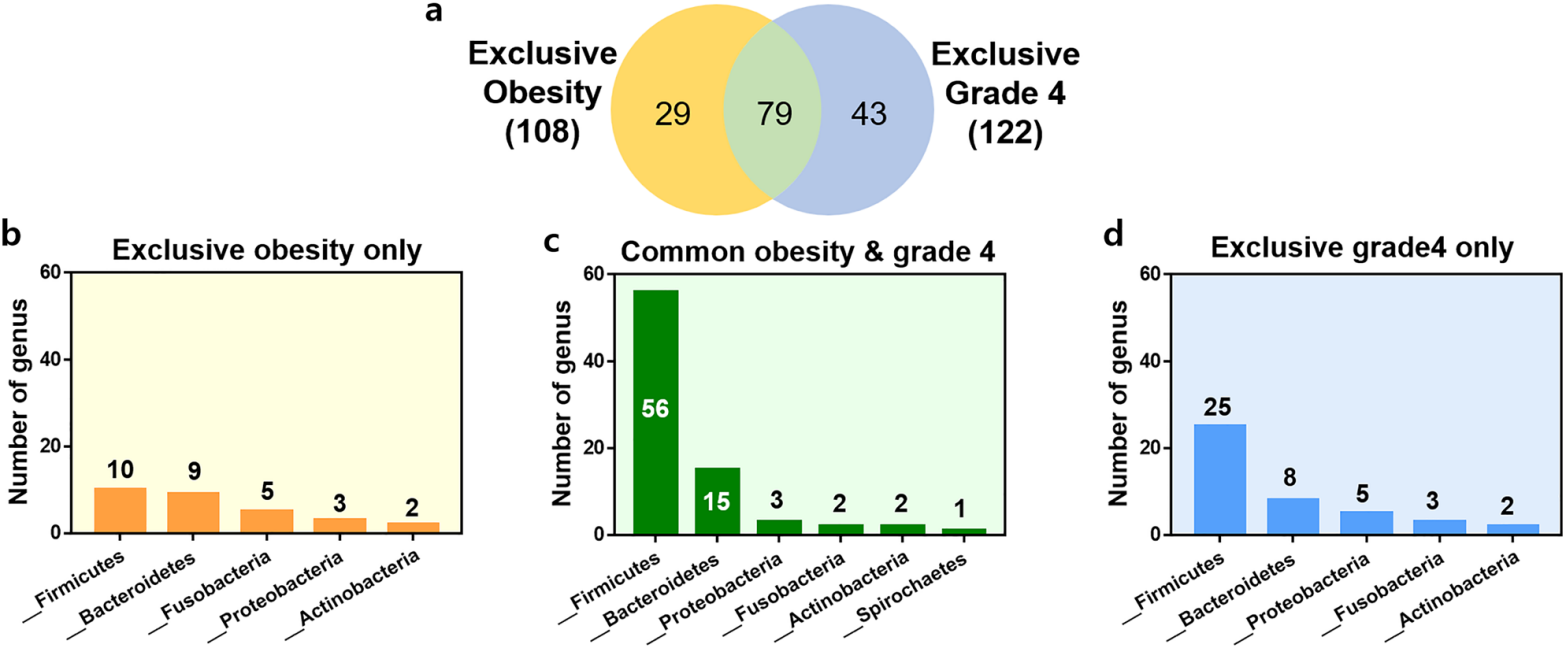
Classification of exclusive ASVs in the obese and grade 4 groups. The Venn diagram shows the overall number of ASVs overlapping between obese-exclusive ASVs in category 1 and grade 4-exclusive ASVs in category 2. The bar plot shows the microbial composition at the genus level of the unique ASVs and common ASVs in each group.

To investigate the association of microbial communities between the obese and tonsil grade groups, the composition of obese-exclusive ASVs in category 1 and grade 4-exclusive ASVs in category 2 was classified using a Venn diagram in Fig. 4. We identified 79 shared ASVs among the obese-exclusive and grade 4-exclusive ASVs, corresponding to 73% of the obese-exclusive and 65% of the grade 4-exclusive ASVs (Fig. 4a). *Firmicutes* dominated the phyla of common ASVs in the Venn diagram, accounting for 70% of the total ASVs (56 out of 79 ASVs) (Fig. 4c). Additionally, *Firmicutes* accounted for 10 out of 29 (34%) ASVs unique to the obese group (Fig. 4b), and 25 out of 43 (25%) ASVs unique to the grade 4 group (Fig. 4d).

### Association between biochemical parameters and bacterial communities of the patients

**Figure 5:**
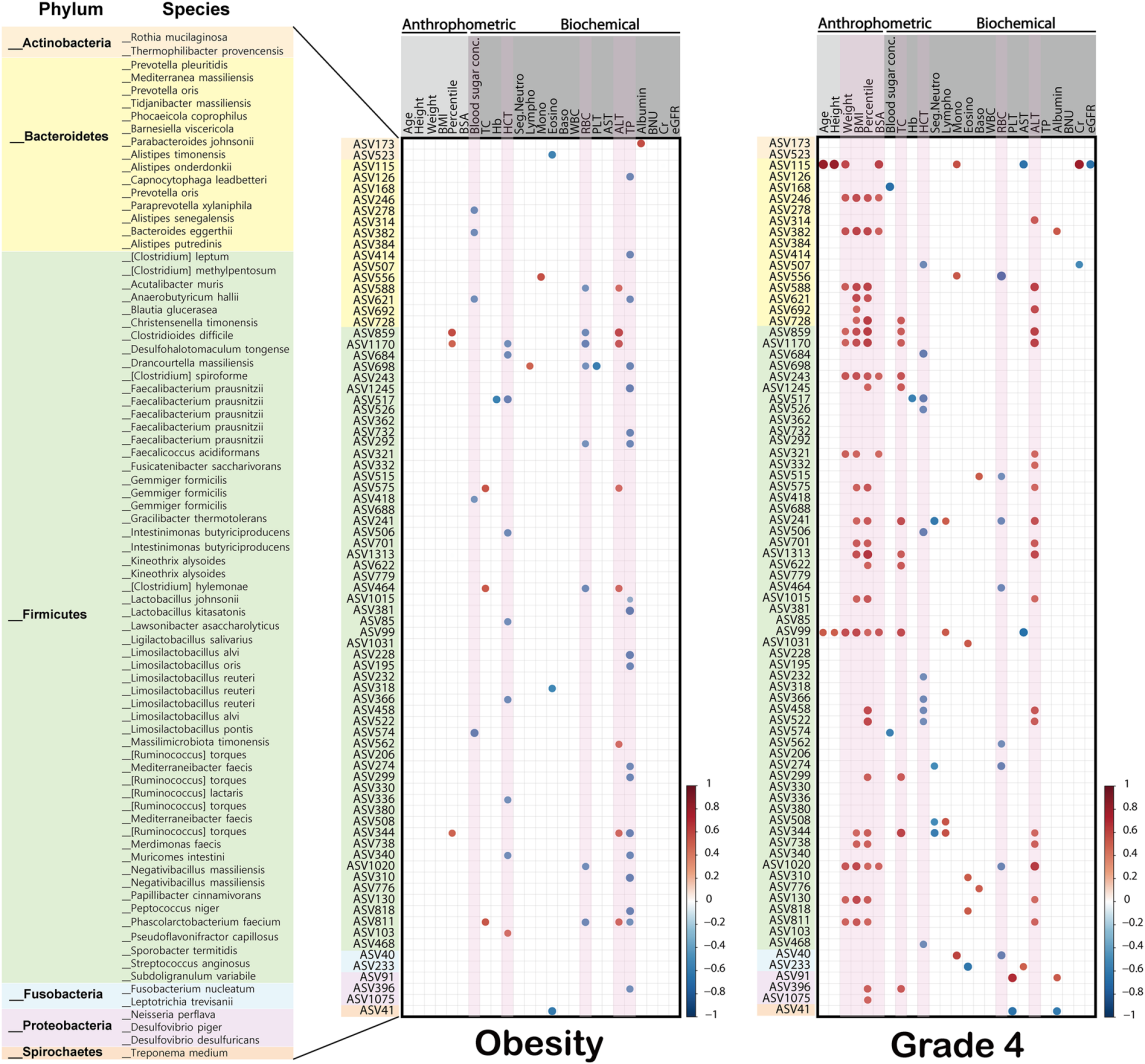
Correlation of biochemical indices and 79 ASVs obtained from overlapping of obese-exclusive ASVs and grade 4-exclusive ASVs. Correlation plot of 79 ASVs in obese and grade 4 groups with 20 biochemical indices and five anthropometric measurements. A correlation matrix plot based on Spearman correlation was developed. Positive and negative correlations are represented by red and blue circles, respectively, and the size and color of the circles refer to the correlation value. All circles show a significant correlation (*P* ≤ 0.05).

We used Spearman correlation analysis to compare the correlations between the 79 common ASVs shared by obese-exclusive and grade 4-exclusive ASVs, using data from 20 biochemical indices and five anthropometric measurements (Fig. 5). Regardless of the group in each category, we observed mostly positive correlations between the ASVs (Fig. S2). More ASVs had a significant correlation with the obese and grade 4 groups than with the lean and grade 2 and 3 groups.

In the obese group, ASVs showed a significant negative correlation with blood sugar concentration, HCT, RBC, and total protein, but a positive correlation with ALT. In the grade 4 group, ASVs showed a significant negative correlation with HCT and RBC, and a significantly positive correlation with weight, BMI, body weight percentile, body surface area, total cholesterol, and ALT. In both the obese and grade 4 groups, HCT and RBC showed a significant negative correlation with ASVs, and ALT showed a significant positive correlation.

## Discussion

To the best of our knowledge, this is the first study to classify and investigate the correlation between the core microbiome of tonsils in Korean pediatric patients and tonsil hyperplasia based on the BMI percentile and the grade of tonsil hyperplasia. The first category of BMI percentile was divided into lean and obese groups based on the most up-to-date Korean National Growth Chart. The second category of tonsil hyperplasia was divided into three groups (grade 2, 3, and 4) using the Brodsky grading scale, classifying the palatine tonsil into five grades with high reliability^21^. All patients enrolled in our study had tonsil hyperplasia as we conducted microbiome analysis on the surgically resected palatine tonsil. Thus, we had no control data for healthy microbiomes as grade 0 and 1 children were excluded from this study. In addition, to exclude the effects of inflammation and antibiotic use^22^, we only enrolled children who had no recent clinical events of inflammation. Thus, our data is clinically significant because we analyzed a relatively homogenous whole palatine tonsillar microbiome cohort, and our study participants maintained a fasting period of at least 12 h before the surgery.

Our data revealed similar microbiome profiles in obese patients and in grade 4 patients with tonsillar hyperplasia (73% and 65% shared unique ASVs in obese and grade 4 hyperplasia, respectively). Moreover, the grade 4 group ASVs were positively correlated with body weight, BMI, percentile of body weight, and body surface area (Figs. 4a and 5), suggesting that the microbiome in enlarged tonsils may also influence the development of obesity. These findings are consistent with results from previous clinical studies and confirm the possible relationship between tonsillar hypertrophy and obesity from a metagenomic point of view^23^.

In a previous study, we examined the microbial communities in the saliva and tonsils of Korean children who underwent tonsillectomy due to tonsil hypertrophy^7^. The study found that the tonsil microbiota was dominated by *Proteobacteria, Firmicutes, and Bacteroidetes*, with the genera *Haemophilus*, *Streptococcus*, and *Fusobacterium, Veillonella and Prevotella* genera being the most abundant in both the tonsils and saliva^24^. Thus, the community structures (*Haemophilus*, *Streptococcus*, and *Fusobacterium*) were preserved regardless of BMI percentile and the grade of tonsil hyperplasia.

Several studies have reported that gut microbiota diversity and abundance differ between obese and non-obese individuals, but the results are inconsistent^25,26^. A microbial community study in Korean children found no significant difference between the two groups, while a study in Korean adults reported lower phylogenetic diversity in the obese group based on BMI^27,28^. Thus, the microbiome diversity in subjects with or without obesity varied depending on the participant age, growth stage, and living environment.

**Figure 6:**
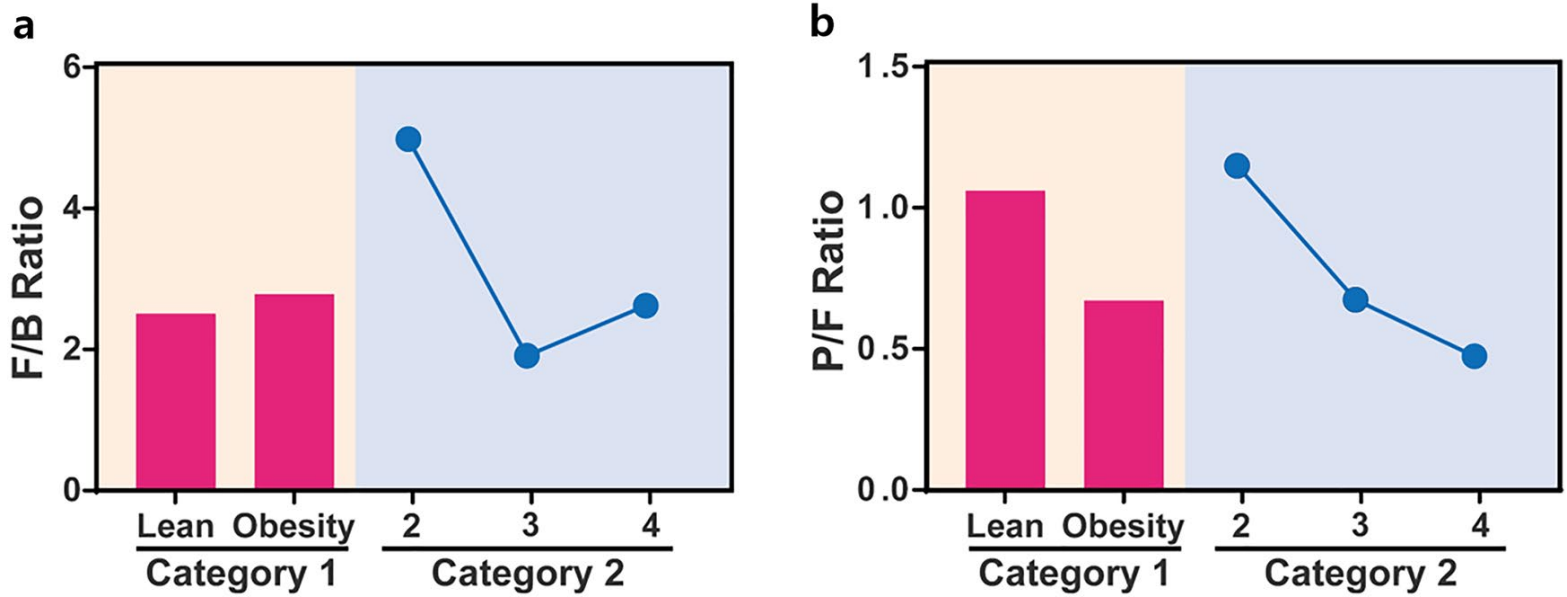
Bacterial phyla ratios by category. Bar plots for category 1 and scatter plots for category 2 show (**a**) Firmicutes to Bacteroidetes ratio and (**b**) Proteobacteria to Firmicutes ratio.

The relative abundances of Firmicutes (F) and Bacteroidetes (B) in the gut microbiome vary significantly across individuals within the same population due to various lifestyle factors including diet, physical activity, and antibiotic consumption^29^. The F/B ratio is an important factor for normal intestinal homeostasis. Several studies indicate that obese people have a higher F/B ratio than those with average weight, suggesting that the gut F/B ratio could serve as an obesity biomarker^30–32^. In particular, the F/B ratio is increased in people with obesity compared to that in people with average weight, and high rates of Firmicutes could serve a role in the development of obesity^33^. While previous studies have indicated that the gut microbiota could contribute to the development of obesity, the evidence linking obesity to alterations in the F/B ratio is unconvincing^28,34,35^. However, the F/B ratio in the oral microbiome remained relatively unexplored. Here, the observed F/B ratios were 2.51 and 2.78 in the lean and obese groups, respectively (Fig. 6a). Previous studies reported an increased F/B ratio in children with snoring or OSA^36^. We observed the highest F/B ratio in the grade 2 group in category 2, which was significantly decreased in grade 3 and 4 groups (4.96 vs. 1.91 and 2.62, respectively) (Fig. 6a). A decreased F/B ratio is associated with inflammatory bowel disease^32^, and our patient group was selected by excluding clinical features of inflammation, thus chronic inflammatory conditions might be partly involved in tonsillar hypertrophy. This result suggests that controlling chronic inflammation in grades 3 and 4 could alleviate symptoms by reducing the tonsil size, highlighting the need for early treatment.

Moreover, to simplify the complexity of microbiota composition, we focused on the two major bacterial phyla in the tonsil microbiome: Proteobacteria and Firmicutes. We observed a consistently decreased abundance of Proteobacteria in the obese and enlarged tonsil groups, with a consistent reduction in the Proteobacteria to Firmicutes (P/F) ratio (Fig. 6b). Although this study was limited to patients with snoring and did not measure OSA objectively, we were able to calculate the P/F ratio using data from previous studies, that confirmed reduced P/F ratio in OSA^37,38^. Proteobacteria is a more abundant phylum than Bacteroidetes in the tonsillar microbiome; however, there is no study on the P/F ratio in the tonsillar microbiome. Thus, further studies are needed to evaluate the significance of the P/F ratio in future oropharyngeal microbiome research, as Firmicutes and Bacteroidetes are the main predominant phyla in the gut microbiome^39^.

A recent study has suggested that OSA and microbiome dysbiosis may have bidirectional associations^40^. Reports have indicated that sleep-related factors are linked to microbiome dysbiosis, and pediatric OSA patients have severely dysregulated microbiomes. A study found a significant association between *Streptococcus pyogenes* and OSA in children^41^. We observed decreased *Proteobacteria* and increased *Fusobacteria* and *Spirochetes* in obese and grade 3 and 4 groups, similar to a previous study that identified *Proteobacteria* in patients with chronic tonsillitis, and *Fusobacteria* and *Spirochetes* in patients with OSA^42^. This study is distinct in that it examined the microbiome of tonsils in the absence of tonsillitis.

We investigated the correlation between 79 shared ASVs in the obesity and grade 4 groups and 25 variables (20 biochemical indices and five anthropometric variables). While there was no clinical difference between hematologic variables such as HCT and RBC for each group of categories 1 and 2, we observed a significant negative correlation between HCT and RBC with ASVs in both categories in the metagenomic analysis. A study using obese mice reported an association between the microbiota and significantly increased blood glucose levels and decreased RBC, HCT, and hemoglobin levels^43^. Moreover, an investigation of the gut microbiome and hematological parameters in Tibetans reported that age, BMI, RBC, hemoglobin, HCT, and platelet count were associated with the structure of the gut microbiome, whereas BMI and platelet count were significant explanatory variables^44^.

Among the various biochemical indices, ALT levels increased with obesity and tonsillar hypertrophy without AST elevation. As reported by Verrijken et al*.*, elevated ALT levels without AST level increase in children with obesity can indicate non-alcoholic fatty liver disease^45^. Although pathologically elevated liver enzyme levels were not found in our pediatric cohort, we cannot give clinical significance to the AST/ALT ratio. The solitary elevation of ALT consistently observed in all categories has clinical relevance in that absolute ALT elevation (with an upper limit of normal) could be an early sign of fatty liver disease, even in overweight or obese children with normal liver enzymes^46^. This association is explainable in that ALT levels in overweight and obese patients were positively correlated with visceral adipose tissues, which has been associated with the pathogenesis of nonalcoholic fatty liver disease, a condition regarded as the hepatic component of the metabolic syndrome^47^. Interestingly, the increase in ALT according to tonsil size grade is a novel finding that requires further investigation. However, it is still understandable that these children underwent surgery mainly because of snoring and mouth breathing, which are related to OSA, a well-known risk factor for various metabolic syndromes, including non-alcoholic fatty liver disease. Recently, Kang et al*.* reported that higher severity OSA is strongly associated with ALT elevation in Korean children with obesity. Many such children undergo surgery due to snoring and mouth breathing, and it seems that OSA and obesity may have synergistic effects on ALT level elevation^48^. We believe that, based on the results of this study, further analysis of the changes in the oral microbiome and serum ALT after tonsillectomy in children in the future will be very interesting.

Furthermore, in our study, among 79 ASVs shared by obesity and tonsillar hypertrophy grade 4 groups, 10 ASVs, and 19 ASVs showed significant positive correlations with serum ALT levels in obesity and tonsillar hypertrophy grade 4 groups, respectively. Our result suggests a possible association of tonsillar microbiome and liver metabolism. There is some evidence connecting elevated serum ALT levels to altered gut microbiome composition. However, no report shows a significant association between serum ALT levels and the tonsillar microbiota. Despite the limitations, our findings demonstrate the association between systemic metabolism and the tonsillar microbiome, highlighting the possible influence of the tonsillar microbiome on the metabolic health of the host.

There are some limitations to this study. First, the patient number in subgroups based on body weight status in grade 2 is biased due to small sample size, which should be considered when interpreting the data (Supplementary Table 1). This imbalance in the ratio of obese to lean children in grade 2 is due to a sharp decrease in the number of pediatric tonsillectomies due to the COVID-19 pandemic. However, we were able to find clinical relevance by focusing on obesity and tonsil grade 4 data, which had a relatively balanced distribution in the enrolled patient numbers. The second limitation is that we did not perform polysomnography to objectively diagnose OSA, because when planning tonsillectomy in pediatric patients, the Korean insurance system does not provide routine PSG testing. However, since there is evidence that tonsil size and OSA severity are well correlated, it is possible that tonsil hypertrophy can be interpreted indirectly as OSA severity^49^. Third, other metabolic markers, including lipid profiles such as triglyceride, low-density lipoprotein cholesterol, high-density lipoprotein cholesterol, and central adiposity using waist circumference were not evaluated. Considering the increasing prevalence of obesity and OSA among children, known synergistic effect of OSA, and obesity on ALT elevation^48^, future studies on the effects of the tonsillar microbiome on dyslipidemia and systemic metabolic syndrome will be clinically relevant. Lastly, this study was cross-sectional and thus unable to conclude the direction of associations or causal effects in the association of obesity and tonsil hyperplasia. The relationships between the tonsillar microbiota and obesity or tonsillar hyperplasia need to be further explored. In addition, future prospective trials on the effects of tonsillectomy or tonsillar microbiome modulation using specific probiotics with a larger sample size will be of interest and provide further understanding of the tonsillar microbiome.

In conclusion, we showed that children with obesity and tonsil hypertrophy have comparable microbiome compositions and induce similar changes in the microbiome abundance and composition, confirming the association between obesity and tonsil hypertrophy from a metagenomic perspective. Moreover, these changes were well correlated with the P/F ratio rather than the F/B ratio, which is a well-accepted dysbiosis marker in the gut. Thus, we identify the P/F ratio as a novel and promising biomarker reflecting oral or tonsil dysbiosis, which should be verified in future studies. Finally, we identify a possible effect of the tonsillar microbiome on metabolic disorders of the host. These results shed light on the importance of dysbiosis of the tonsil and suggest the need for its regulation, especially during childhood.

## Methods

### Ethic approval and consent to participants

This study was approved by the Institutional Review Board of Chungnam National University Hospital (IRB No. CNU2018-06-021-002) and conducted in accordance with the Declaration of Helsinki (1989) by the World Medical Association. The Institutional Review Board of Chungnam National University Hospital waived the requirement for informed consent due to the anonymity of the information.

### Sample collection

We enrolled 46 out of 66 patients who underwent tonsillectomy at Chungnam National University Hospital (CNUH, Daejeon, Korea) between June 2018 and August 2021. Selection criteria included age ˂ 10 years and presence of benign tonsillar hypertrophy with a grade ≥ 2 on the Brodsky grading scale, leading to sleep apnea with or without mouth breathing. Patients with a history of recent (within the last 3 months) or frequent (3 times/year) tonsillitis, systemic disease, antibiotic intake within the last 3 months, or regular use of oral rinse were excluded.

As shown in Supplementary Table 1, the 46 participants were classified into two categories: category 1, based on the 85th body mass index (BMI) percentile by sex and age according to the 2017 Korean National Growth Charts for Children and Adolesecents^20^, the patients were divided into lean and obese groups, with 28 patients classified as lean and 18 as obese; category 2, the patients were classified into three groups of grades 2, 3, and 4, based on tonsil size measured according to the Brodsky grading scale, with 12, 18, and 16 patients, respectively. Using two-way analysis of variance (ANOVA) to estimate sample size for the two categories of groups, the overall power with a large effect size (*f* = 0.4) was 0.8 at a significance level of 0.05 (Statsmodels 0.14.0 in Python).

Oral examination and sample collection were conducted at the Department of Otolaryngology-Head and Neck Surgery of CNUH. All participants were required to fast after midnight the day before the surgery, according to standard preoperative protocol. Saliva was collected from the mouths of the patients on the morning of the surgery. Participants were not allowed to eat or drink for at least nine hours prior to sample collection and were instructed not to brush their teeth the previous night, until the time of sampling. Then, the participants were instructed to chew on a sterile gauze (4 × 4 in.) for 1 min to induce salivation, before collecting the gauze containing saliva in a sterile Eppendorf conical tube (50 mL). The tubes were centrifuged at 1763×*g* for 15 min, with a 100 μm cell strainer to collect clear saliva samples, which were immediately stored at – 70 °C, until DNA extraction.

Following the administration of general anesthesia, *en bloc* resection of both the palatine tonsils was performed via the peroral approach using a cold knife and electrocautery, as per the standard tonsillectomy protocol. The tissue around the upper pole of the palatine tonsils was sent to a pathology laboratory for clinical diagnosis, whereas the other half was used for microbiome analysis.

### Anthropometric measurements of subjects

The patients were divided into two categories. The first category was divided into two groups based on obesity status according to the 85th percentile with reference to “the 2017 Korean National Growth Chart”^20^. The second category was divided into three groups with tonsil grades 2, 3, and 4, based on the Brodsky grading scale^50^. For each child, three independent observers of various seniority levels (staff otolaryngologist and senior or junior resident) assessed and measured the tonsil size preoperatively by visual inspection, using the Brodsky scale depending on the percentage of oropharyngeal conduit occupied by the tonsil as follows: grade 0 indicated previous tonsillectomy; grade 1 showed that the tonsils were hidden in the pillars; grade 2 indicated that the tonsils were beyond the anterior pillar and between 25 and 50% of the pharyngeal space; grade 3 indicated that the tonsils were beyond the pillars but not up to the middle and occupied > 50% and up to 75% of the pharyngeal space; grade 4 indicated that the tonsils occupied > 75% of the pharyngeal space.

Anthropometric data was collected for all participants, including height, weight, and body surface area, measured the day before the surgery. The BMI was determined as previously described^51^.

### Serum biochemical indices of the patients

According to the guidelines of the American Society of Anesthesiologists, routine preoperative laboratory tests were conducted within 2 weeks prior to surgery to obtain serum biochemical indices, including blood glucose, white blood cell, red blood cell (RBC), and platelet counts, hemoglobin, hematocrit (HCT), segmented neutrophils, lymphocytes, monocytes, eosinophils, basophils, total protein, albumin, blood urea nitrogen, creatinine, estimated glomerular filtration rate, total cholesterol, and the liver enzymes, aspartate aminotransferase (AST) and alanine aminotransferase (ALT).

### Library construction and sequencing

DNA was extracted using a DNeasy PowerSoil Kit (Qiagen, Hilden, Germany), as per the manufacturer's instructions. The extracted DNA was quantified using a Quant-IT PicoGreen kit (Invitrogen, Eugene, OR, USA). Sequencing libraries were prepared according to Illumina 16S Metagenomic Sequencing Library protocols to amplify the V3 and V4 regions. The input gDNA (2 ng) was PCR-amplified using 5× reaction buffer, 1 mM of dNTP mix, 500 nM each of universal F/R PCR primer, and Herculase II fusion DNA polymerase (Agilent Technologies, Santa Clara, CA, USA). The parameters for the first PCR cycle were heat inactivation for 3 min at 95 °C, followed by 25 cycles of 30 s at 95 °C, 30 s at 55 °C, and 30 s at 72 °C, followed by a 5 min final extension at 72 °C. The universal primer pair with Illumina adapter overhang sequences used for the first amplification was as follows: V3-F: 5′-TCGTCGGCAGCGTCAGATGTGTATAAGAGACAGCCTACGGGNGGCWGCAG-3′, V4-R: 5′-GTCTCGTGGGCTCGGAGATGTGTATAAGAGACAGGACTACHVGGGTATCTAATCC-3′. The first PCR product was purified using AMPure beads (Agencourt Bioscience, Beverly, MA, USA). Following purification, 2 µl of the first PCR product was further PCR-amplified for final library construction containing the index using NexteraXT Indexed Primer. The conditions for the second PCR cycle were the same as that for the first PCR cycle, with 10 cycles instead of 25. The PCR products were purified using AMPure beads. The final purified product was quantified using qPCR according to the qPCR quantification protocol guide (KAPA library quantification kits for Illumina sequencing platforms) and qualified using TapeStation D1000 ScreenTape (Agilent Technologies, Waldbronn, Germany). Paired-end (2 × 300 bp) sequencing was performed by Macrogen using the MiSeq System (Illumina, San Diego, USA).

### Analysis of bacterial compositions of microbiomes

The tonsillar tissues of 46 participants generated a total of 4,029,186 reads, with an average of 87,591 reads and a standard deviation of 11,284 reads. Paired-end reads matching the adapter sequences were trimmed using the Cutadapt software^52^. Quality filtering, denoising, merging, and chimera removal steps were conducted using DADA2, resulting in a total of 3,114,087 merged reads. A total of 4778 amplicon sequence variants (ASVs) produced by DADA2 shared with taxonomic assignments were generated using the BLAST database.

### Statistical analysis

Alpha diversities were calculated from a rarefied dataset using the QIIME pipeline. The alpha diversities for species richness and evenness (Chao1 and Shannon indices, respectively) were calculated. Statistical comparison of the alpha diversities within the percentile body weight (category 1: lean and obese) and tonsil size (category 2: grades 2, 3, and 4) groups was performed using the Kruskal–Wallis test. The count data were then transformed to centered log ratios (CLR) using the microbiome v1.16.0 library (option: transform = “clr”) in R v4.1.2. The transformed matrix was then ordinated with Aitchison distance and plotted on a principal component analysis (PCA). To statistically evaluate the significance of grouping, analysis of similarities (ANOSIM) was performed using the R package “vegan” (distance = “Euclidean,” Permutations = 999).

### Supplementary Information

Supplementary Information.

## Data Availability

Raw sequences have been deposited in the NCBI Sequence Read Archive (SRA) repository (BioProject accession no. PRJNA907188).
