# Supplementary material for: The association of tonsillar microbiota with biochemical indices based on obesity and tonsillar hypertrophy in children
Source: Sci Rep. 2023 Dec 20;13:22716. doi: 10.1038/s41598-023-49871-y (PMC10733282; doi:10.1038/s41598-023-49871-y)
Supplement: Supplementary file 1 — Supplementary Information. [file 41598_2023_49871_MOESM1_ESM.pdf]

Supplementary Figures:

Supplementary Figure. 1 The rarefaction curves for (a) category 1 and (b) category 2

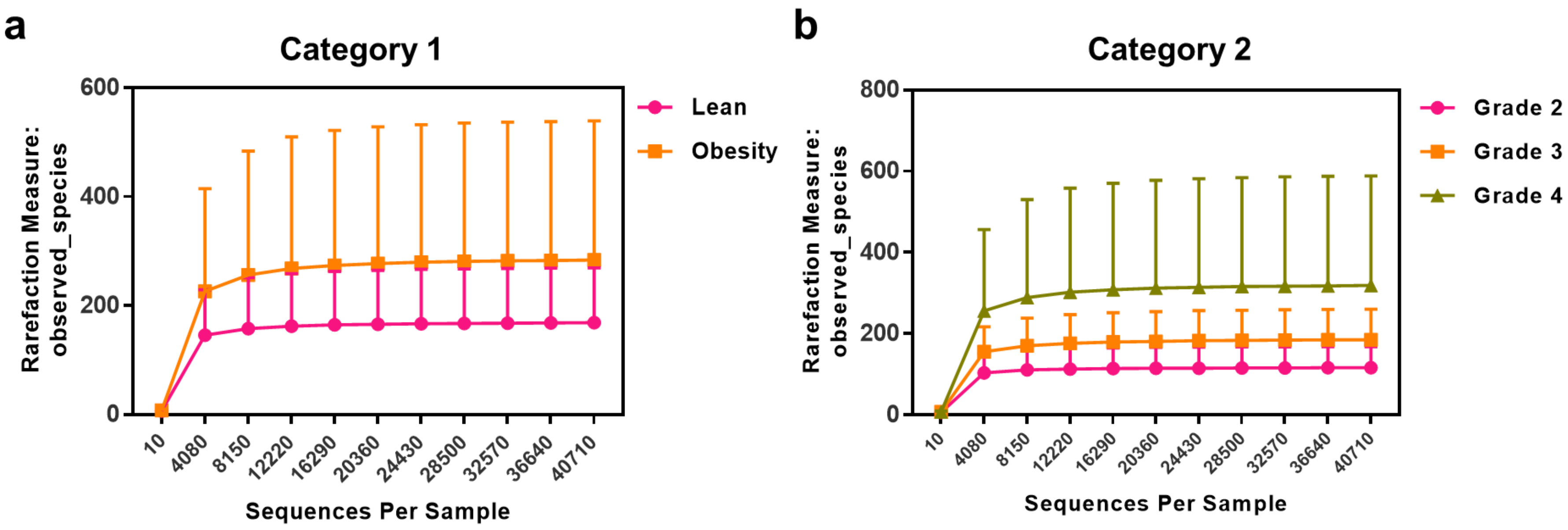

Supplementary Figure. 2 The correlation plot of the biochemical indices and 79 ASVs obtained from overlapping of obese-exclusive ASVs and grade 4-exclusive ASVs. A correlation matrix plot based on Spearman correlation was developed. Positive and negative correlations are represented by red and blue circles, respectively, and the size and color of the circles refer to the correlation value. All circles show a significant correlation ($P \leq 0.05$).

[Category 1]

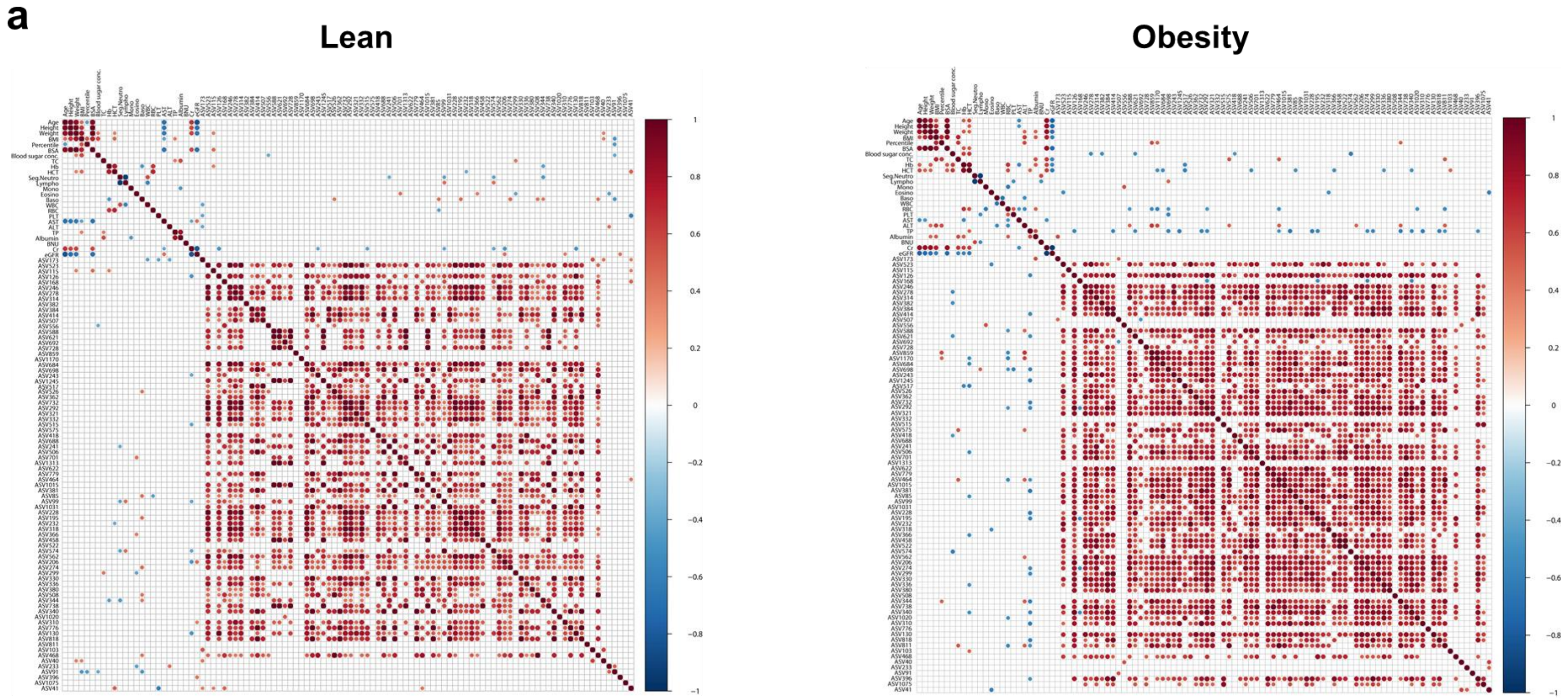

[Category 2]

b

Grade 2 **Grade 3** **Grade 4**

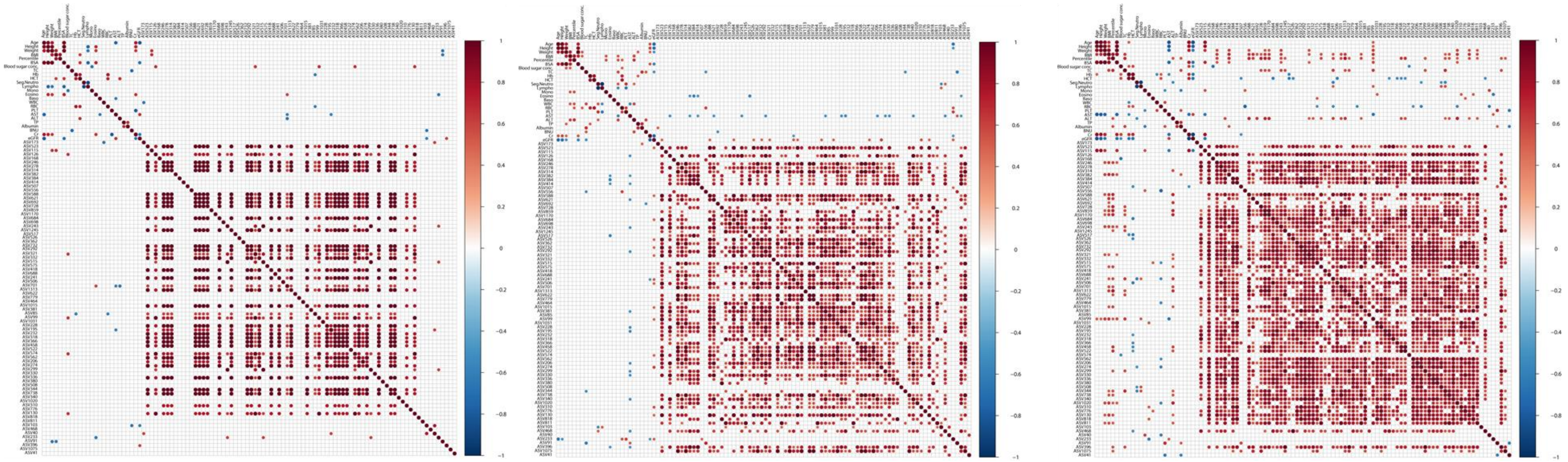

Supplementary Tables:

Supplementary Table 1. Number of patients in each group by category

The 46 patients were divided into two categories: category 1 was classified into lean or obese groups based on the 85th BMI percentile by sex and age based on the 2017 Korean National Growth Chart, while category 2 was classified with grades 2, 3, and 4, based on tonsil size.

		Category 2			
		Grade 2	Grade 3	Grade 4	Total
Category 1	Lean	11	10	7	28
	Obesity	1	8	9	18
	Total	12	18	16	46

Supplementary Table 2. General characteristics and biochemical indices of participants.

Anthropometric measurements and biochemical indices in this study, for each category, are represented as the mean ± standard deviation, and the reference value range for Korean children. Differences in each category were analyzed using the unpaired *t*-test and one-way analysis of variance, followed by Tukey's post hoc test.

Parameters	Total (Mean ± S.D.)	Category 1			Category 2				Korean children
		Lean (Mean ± S.D.)	Obesity (Mean ± S.D.)	p-value	Grade 2 (Mean ± S.D.)	Grade 3 (Mean ± S.D.)	Grade 4 (Mean ± S.D.)	p-value	
**Anthropometric measurement**									
**Boys (N)**	33	20	13		9	13	11		
**Girls (N)**	13	8	5		3	5	5		
**Age (years)**	7.89 ± 2.68	7.5 ± 2.40	8.5 ± 3.05		6.58 ± 1.62	8.17 ± 2.07	8.56 ± 3.6		6~12
**Height (cm)**	129.65 ± 17.31	127.55 ± 15.50	132.92 ± 19.83	0.31	121.63 ± 10.71	131.96 ± 14.65	133.08 ± 22.39	0.064	114.7~151.7
**Weight (kg)**	33.48 ± 15.31	27.59 ± 9.85	42.65 ± 17.90	0.004	24.14 ± 5.70	34.76 ± 12.14	39.05 ± 20.19	0.002	20.7~45.4
**Body weight percentile**	62.41 ± 32.60	41.07 ± 23.48	95.61 ± 4.55	<0.001	46.25 ± 28.54	64.11 ± 32.53	72.63 ± 32.68	0.066	
**BMI (kg/m^2)**	18.95 ± 4.18	16.41 ± 1.82	22.89 ± 3.73	<0.001	16.09 ± 1.66	19.46 ± 4.15	20.51 ± 4.62	<0.001	15.8~19.8
**BSA (m^2)**	1.10 ± 0.32	1.00 ± 0.25	1.25 ± 0.36	0.012	0.95 ± 0.22	1.12 ± 0.26	1.19 ± 0.41	0.187	
**Blood pressure (mmHg)**	120.30 ± 11.58 /74.79 ± 10.17	120.12 ± 9.97 /74.76 ± 9.43	120.56 ± 13.81 /74.83 ± 11.40	0.767 /0.824	120.20 ± 9.21 /73.20 ± 12.39	120.06 ± 10.66 /74.06 ± 8.57	120.63 ± 14.25 /76.56 ± 10.65	0.992 /0.706	
**Biochemical indices**									
**Blood glucose (mg/dL)**	93.65 ± 11.81	93.25 ± 13.37	94.28 ± 9.20	0.777	90.08 ± 16.37	93.94 ± 7.67	96 ± 11.78	0.594	70~115
**Total cholesterol (mg/dL)**	158.65 ± 41.01	161.80 ± 35.58	154.14 ± 50.46	0.782	171.6 ± 26.31	159.67 ± 41.59	162.08 ± 25.29	0.663	< 170
**Hemoglobin (g/dL)**	13.38 ± 0.68	13.30 ± 0.49	13.49 ± 0.92	0.317	13.08 ± 0.65	13.36 ± 0.6	13.4 ± 0.83	0.440	

**Hematocrit (%)**	39.95 ± 1.94	40.02 ± 1.50	39.86 ± 2.58	0.178	37.63 ± 1.82	39.23 ± 2.03	39.73 ± 2.18	0.029	
**Segmented Neutrophils (%)**	50.82 ± 5.91	48.70 ± 10.13	53.86 ± 6.30	0.610	45.07 ± 6.04	48.07 ± 8.24	51.49 ± 8.28	0.103	
**Lymphocytes (%)**	39.55 ± 9.76	41.60 ± 11.37	36.61 ± 6.54	0.502	44.04 ± 7.31	42.91 ± 8.33	39.09 ± 8.38	0.229	
**Monocytes (%)**	6.29 ± 1.33	6.00 ± 1.61	6.70 ± 0.70	0.014	5.78 ± 1.69	5.57 ± 1.22	6.27 ± 1.41	0.359	
**Eosinophils (%)**	2.74 ± 1.22	3.08 ± 1.32	2.24 ± 0.95	0.301	4.52 ± 2.46	2.91 ± 1.45	2.49 ± 2.1	0.034	
**Basophils (%)**	0.61 ± 0.47	0.61 ± 0.26	0.61 ± 0.70	0.607	0.62 ± 0.19	0.53 ± 0.26	0.67 ± 0.48	0.442	
**AST ($\times 10^6$/uL)**	25.71 ± 5.62	25.70 ± 5.42	25.71 ± 6.34	0.231	25.25 ± 3.36	22.17 ± 5.28	27.81 ± 12.95	0.07	15~40 (Boys), 13~35 (Girls)
**ALT ($\times 10^3$ /ul)**	13.88 ± 4.81	11.10 ± 2.69	17.86 ± 4.41	<0.001	12.75 ± 3.93	13.5 ± 5.34	20.56 ± 11.93	0.044	10~35 (Boys), 10~30 (Girls)
**AST/ALT ratio**	1.86 ± 0.75	2.21 ± 0.67	1.31 ± 0.51	<0.001	2.12 ± 0.56	1.89 ± 0.84	1.62 ± 0.74	0.153	
**WBC ($\times 10^3$/mL)**	6.73 ± 1.95	6.56 ± 1.57	6.97 ± 2.52	0.521	7.35 ± 2.82	7.01 ± 1.78	6.78 ± 2.03	0.9	4.5~13.5
**RBC ($\times 10^6$/mL)**	4.81 ± 0.23	4.78 ± 0.20	4.85 ± 0.27	0.494	4.7 ± 0.32	4.78 ± 0.25	4.74 ± 0.23	0.696	4.2~6.0
**PLT ($\times 10^3$/mL)**	321.53 ± 79.84	319.10 ± 100.55	325.00 ± 42.55	0.883	308.67 ± 53.52	330.33 ± 77.99	272.13 ± 46.3	0.032	150~350
**Total protein (n)**	6.96 ± 0.39	6.91 ± 0.42	7.04 ± 0.34	0.298	6.86 ± 0.35	7.05 ± 0.46	6.94 ± 0.34	0.419	
**Albumin**	4.29 ± 0.22	4.27 ± 0.23	4.32 ± 0.20	0.462	4.25 ± 0.19	4.32 ± 0.24	4.28 ± 0.22	0.656	
**BUN**	12.36 ± 3.15	11.81 ± 2.62	13.22 ± 3.76	0.140	11.49 ± 1.88	12.83 ± 4.07	12.49 ± 2.75	0.367	
**Cr**	0.43 ± 0.12	0.41 ± 0.07	0.45 ± 0.17	0.778	0.37 ± 0.04	0.42 ± 0.09	0.48 ± 0.17	0.029	
**eGFR**	325.40 ± 104.86	330.07 ± 91.01	318.38 ± 125.31	0.719	382.89 ± 69.44	316.89 ± 106.43	289.6 ± 112.75	0.061	
